# Mechanism of spacer integration links the CRISPR/Cas system to transposition as a form of mobile DNA

**DOI:** 10.1186/s13100-015-0039-3

**Published:** 2015-05-12

**Authors:** Fred Dyda, Alison B Hickman

**Affiliations:** Laboratory of Molecular Biology, National Institute of Diabetes and Digestive and Kidney Diseases, National Institutes of Health, 5 Center Dr., Bethesda, MD 20892 USA

**Keywords:** CRISPR/Cas, Adaptive immunity, Cas1 nuclease protein, DNA transposition, Integrase

## Abstract

It has recently become clear that many bacterial and archaeal species possess adaptive immune systems. These are typified by multiple copies of DNA sequences known as clustered regularly interspaced short palindromic repeats (CRISPRs). These CRISPR repeats are the sites at which short spacers containing sequences of previously encountered foreign DNA are integrated, and the spacers serve as the molecular memory of previous invaders. *In vivo* work has demonstrated that two CRISPR-associated proteins - Cas1 and Cas2 - are required for spacer integration, but the mechanism by which this is accomplished remained unclear. Here we review a recent paper describing the *in vitro* reconstitution of CRISPR spacer integration using purified Cas1 and Cas2 and place the results in context of similar DNA transposition reactions and the crystal structure of the Cas1/Cas2 complex.

## Background

**Figure 1:**
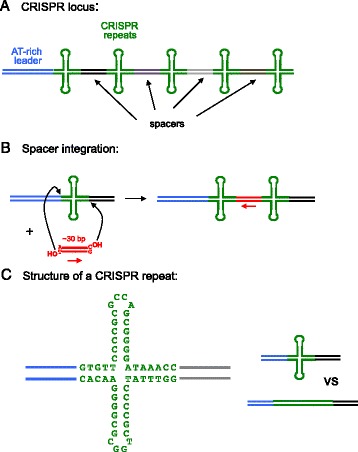
Overview and details of a CRISPR locus. **(A)** Clustered regularly interspaced short palindromic repeats (CRISPRs, in green) typically alternate with spacers of different sequence but similar length (shades of gray and black). **(B)** Proposed double-ended integration of a protospacer (red) into the site of a CRISPR repeat. This results in a duplication of the CRISPR. **(C)** Sequence and deduced cruciform structure of the *E. coli* 28 nt CRISPR repeat.

The clustered regularly interspaced short palindromic repeats (CRISPR)/CRISPR-associated (Cas) system, present in approximately 90% of archaea and approximately 50% of bacteria, is an adaptive immune system (reviewed in [1]) that generates small RNAs (known as crRNAs) transcribed from chromosomally integrated foreign DNA fragments and uses them to direct the degradation of invading DNA that contains the same sequence. The foreign DNA fragments, called ‘spacers’, are integrated into a chromosomal CRISPR locus composed of an array of short palindromic repeats forming a repeat-spacer-repeat pattern (Figure 1A). Adjacent to the CRISPR locus is the several hundred base pairs long AT-rich ‘leader sequence’ preceded by the *cas* genes. The *cas* genes associated with CRISPR systems are quite variable, with over 45 different gene families identified in various organisms. Although six *cas* genes (*cas1* to *6*) are extensively conserved, only *cas1* and *cas2* are always present in those genomes that contain a CRISPR locus.

The phenomenon of CRISPR/Cas immunity can be divided into three processes: adaptation, crRNA biogenesis, and crRNA-based interference. In adaptation, a 30- to 40-bp fragment of foreign DNA is integrated orientation-specifically at the DNA palindrome next to the leader sequence in a process that duplicates the palindrome such that the spacer is flanked by two of them after integration (Figure 1B). Pre-crRNA is produced as a long transcript from the promoter in the leader and subsequently matured into short crRNAs. The exact process of crRNA biogenesis differs significantly in the three different major types of CRISPR/Cas systems. Target destruction is also mediated by very different protein players in the three major CRISPR/Cas types. Large and complex nucleoprotein assemblies are involved that organize base pairing between crRNA with one strand of a partially melted complementary foreign DNA. It is the type II systems that use Cas9 for targeting and destruction that are currently the best understood.

Although Cas1 and Cas2 are necessary and sufficient for the insertion of a protospacer in CRISPR loci *in vivo* [2], the details of the mechanism have remained unclear. A recent report by Nuñez *et al*. [3] describes the *in vitro* reconstitution of protospacer insertion, shedding light onto many aspects of adaptation.

## Main text

The recent *in vitro* reconstitution of the crucial spacer integration step using purified Cas1 and Cas2 proteins, synthetic oligonucleotides, and plasmid substrates represents a major step forward in defining the fundamental biochemistry of the process [3]. In the presence of divalent metal ions (Mg^2+^ or Mn^2+^), Cas1 and Cas2 were sufficient to integrate 33-bp protospacers into supercoiled CRISPR-containing plasmid substrates. Although Cas2 was not absolutely required, it greatly enhanced the reaction. The terminal 3′-OH of the protospacer was critical, and the products obtained were very similar to those seen during *in vitro* DNA transposition and retroviral integration reactions. Furthermore, the data were consistent with integration occurring via a direct nucleophilic attack by the protospacer terminal 3′-OH on the target scissile phosphate without the involvement of tyrosine or serine residues located in the Cas1 active site. It was also demonstrated that, similarly to retroviral integrases [4], Cas1 could catalyze a disintegration reaction in which it was able to release a protospacer that had been integrated at one of its ends from a target DNA molecule. Cas2 did not enhance disintegration but it did not inhibit it either.

One surprising finding was the appearance of an unexpected product, not observed in known DNA transposition and retroviral integration reactions, that the authors termed ‘Band X’. This turned out to be several products most closely resembling topoisomers of the target supercoiled plasmid. Significantly, none of these products contained integrated protospacers. The authors suggested as a possible explanation that products are the result of one-ended protospacer integration followed by disintegration that reseals the target plasmid in a series of partially relaxed topoisomers, indicative of reversability. While this is reasonable, the observed products were also consistent with simple nicking and resealing of the target plasmid by Cas1 and Cas2. While Cas1 alone can catalyze disintegration, the formation of Band X seemed to require both Cas1 and Cas2.

Most DNA transposition systems generate target site duplications (TSDs) of a characteristic length that is a property of the particular system. TSDs arise as the two 3′-OH groups at the transposon ends (one on the top strand and one on the bottom) are joined to target DNA with a few base pair stagger during double-ended integration. The precise number of base pairs is dependent on the distance between the active sites of transposase molecules in the multimeric transpososome that always contains at least a dimer of the transposase. Similarly, CRISPR spacer acquisition *in vivo* results in duplication of the CRISPR repeat in the target, again suggesting double-ended integration (where the ends correspond to the two ends of a single protospacer). In the *Escherichia coli* system studied, this is equivalent to generating 28 bp TSDs, which is rather long when compared to TSDs commonly observed with DNA transposons. However, the physical distance between the points of the attacks could be much shorter than implied by 28 bp of B-form dsDNA (which would be approximately 90 Å) because of the likely hairpin structure of the CRISPR repeat (Figure 1C).

While the reported *in vitro* system seems efficient in generating single-ended integrations, it is much less so in generating double-ended ones. Generating efficient double-ended integrations in an *in vitro* reconstituted system is not always easy, as has been seen in the early work on retroviral integrases, for instance [5]. Furthermore, *in vitro*, the presence of the CRISPR locus in the supercoiled target plasmid is not absolutely necessary to observe integration events. Nevertheless, if the CRISPR locus is cloned into a pUC19 backbone, it is the preferred site of integration, attracting 71% of all integrations. A further indication that the *in vitro* system recapitulates the fundamental properties of the system is that integrations that did go into the CRISPR locus showed a preference for the leader proximal repeat. There was also a clear orientation bias, as 73% of the integrations into the CRISPR locus showed integration of the 3′ terminal C of the protospacer into the target (−) strand, and not the 3′ T of the opposite strand (Figure 1B). This is significant since the presence of corresponding 5′ G of the spacer is important to establish the AAG protospacer-adjacent motif (PAM), which is critical for crRNA interference to work in *E. coli*.

**Figure 2:**
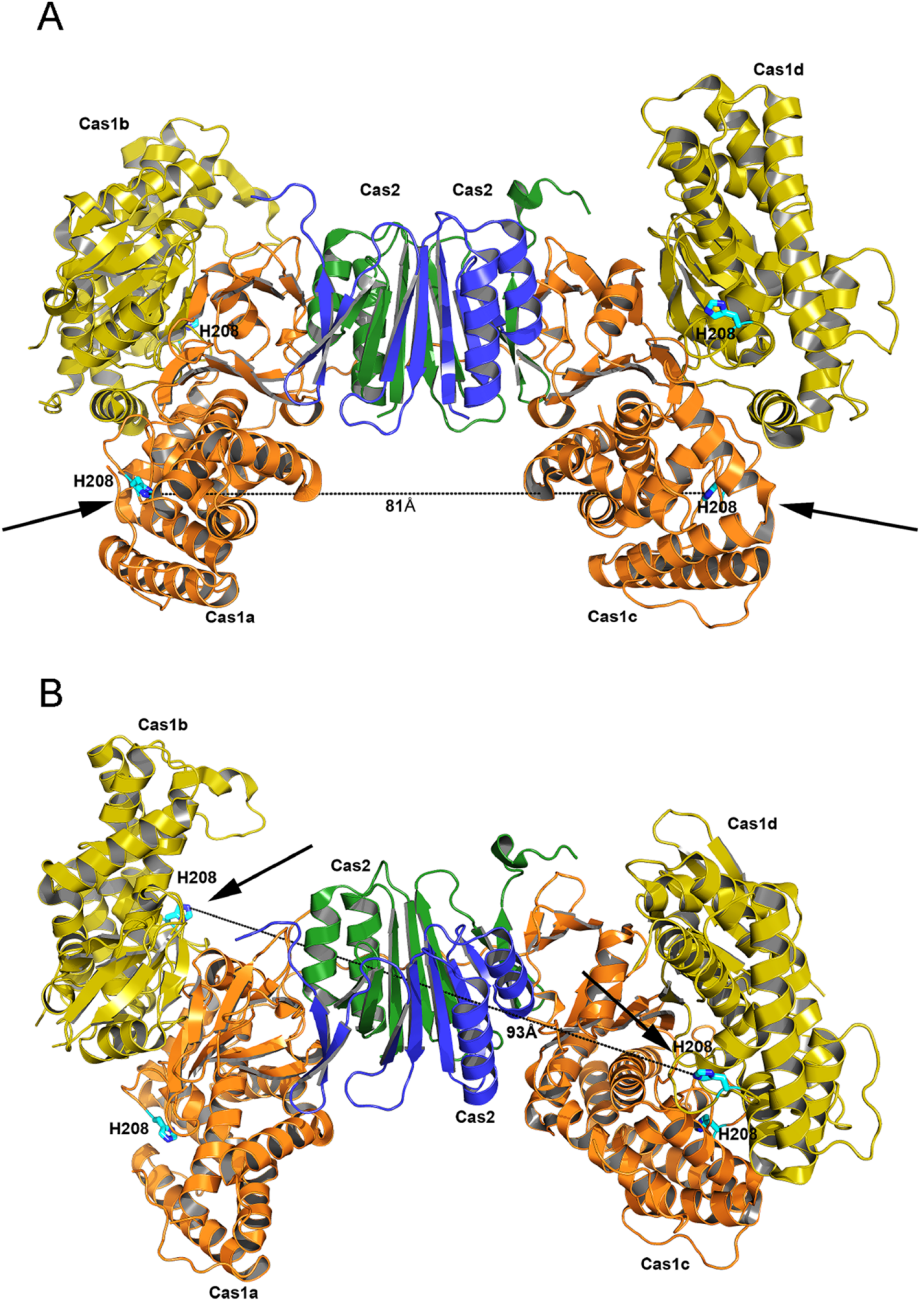
Structure of the Cas1/Cas2 complex (PDB code 4P6I). Cas1 protomers (labelled as in [7]) are shown in gold and orange, the central Cas2 dimer in blue and green. **(A)** Arrows show the active sites (marked by His208, shown as blue sticks) of the two Cas1 protomers that bind directly to the Cas2 dimer. The distance between the active sites, which point away from each other, is approximately 81 Å. **(B)** Horizontal rotation of the view of the complex assembly reveals that the active sites of the other two Cas1 protomers in the heterohexamer are more convincingly pointed towards each other (as expected if these active sites mediate coordinated integration of a protospacer) and are separated by approximately 93 Å. However, the as-the-crow-flies connection between these two active sites passes directly through the intervening Cas2 dimer.

Taken together, the features of the reconstituted *in vitro* system established in [3] are consistent with *in vivo* data and display strong similarities to DNA transposition. However, the protein architectures of neither Cas1 nor Cas2 conform with the paradigms of those known DNA transposases that use direct nucleophilic attack, as they are not members of the retroviral integrase superfamily and do not use three carboxylate side chains (the so-called DDE/D motif) as active site residues. Rather, Cas1 has a unique fold and an active site residue constellation of conserved E, N, H, and D/E residues [6] (Figure 2A). The E/H/D triad has been observed to coordinate a divalent metal ion [6], and mutation of the D abolished the nonspecific nuclease activity of Cas1 [6]. Reassuringly, mutations of the metal-coordinating H and D residues in Cas1 abolished protospacer integration *in vitro*, implicating the involvement of Cas1’s active site.

The role of Cas2 is less clear. It has been reported to be an endonuclease that can act on a variety of substrates [6,7]; however, mutations of active site residues that are important for these activities do not appear to have an effect on spacer acquisition either *in vivo* or *in vitro* [3,7]. Cas1 and Cas2 have been shown to bind to each other, and mutations in the interface that prevent protein-protein interaction were defective in spacer acquisition *in vivo* [7]. Adding to the complexity is that Cas2 is an obligatory dimer, so it is possible that the relevant assembly is a heterotetramer with the Cas2 dimer in the middle with two Cas1 protomers bound on either side (Figure 2B). However, in the crystal structure of the Cas1/Cas2 complex [7], there are two additional Cas1 monomers attached to the heterotetramer through a Cas1/Cas1 interface, making a heterohexameric assembly (Figure 2B). While this assembly is not supported by solution data, the Cas1/Cas1 dimer is twofold symmetric, as usually observed for biologically relevant dimers.

By analogy to DNA transposition reactions, double-ended integration of the protospacer would presumably require two Cas1 active sites. In the crystal structure of the Cas1/Cas2 complex, the distance between the active sites of the Cas1 protomers bound directly to the Cas2 dimer is 81 Å. This is long, but if there is some unfolding of the CRISPR hairpin, it is not unreasonable. In such a model, the CRISPR repeat could be bound by the central Cas2 dimer (Figure 2), where it would possess a dual role as a target binder and dimerization scaffold, neither of which would require endonuclease activity. The difficulty with this view is that these two Cas1 active sites point away from each other so it is very difficult to see how the two 3′-OH groups of an approximately 30-bp protospacer could be simultaneously bound to these active sites. On the other hand, one could also envisage a path that DNA could take between the active sites of the two ‘outer’ Cas1 protomers in the heterohexamers (assuming these exist *in vivo* and not only in crystal structures) as these active sites at least do not point away from each other. Still, with the approximately 93-Å distance between these, it becomes more difficult to imagine how these two could carry out a double-ended integration event of approximately 30-bp-long protospacers.

## Conclusions

The currently available data strongly indicate that CRISPR spacer acquisition is a form of DNA mobility with intriguing similarities to transposition yet employing protein architectures and complex assemblies distinct from what has been seen before in transposition. There is yet another link between CRISPR systems and transposition: recently, a family of Cas1 proteins not associated with CRISPR loci was discovered whose members are encoded by the hypothetical mobile elements designated as casposons [8]. This led to the proposal that parts of the CRISPR system may have their evolutionarily origin in a mobile genetic element [8,9], much like the RAG1 recombinase (involved in the adaptive immune system of vertebrates) originated from the transposase of a eukaryotic *Transib* transposon [10]. The notion is provocative that a Cas1 protein might act as a transposase [11], perhaps alone or in complex with some other currently unidentified partner or partners.

As currently no structural information is available about how the Cas1/Cas2 complex interacts with DNA, there is a lot that has to be left to the imagination. It is unclear how orientation specificity and leader recognition is accomplished, as these imply some breakdown of the twofold symmetry of the Cas1/Cas2 complex. Perhaps one thing is almost certain: a number of big surprises are waiting as was the case when the first DNA transpososome structures became available [12].

## Abbreviations

Cas: CRISPR-associated
CRISPR: clustered regularly interspaced short palindromic repeats
PAM: protospacer-adjacent motif
TSD: target site duplication
